# Spatial control of NLRP3 inflammasome assembly by membrane lipid composition

**DOI:** 10.1038/s42003-026-10541-0

**Published:** 2026-06-23

**Authors:** Paras K. Anand, Najd M. Aljadeed

**Affiliations:** Department of Infectious Disease, Imperial College London, London, UK

**Keywords:** Immunology, Cell biology

## Abstract

The NLRP3 inflammasome has been shown to assemble on multiple organelles, including the mitochondria, endoplasmic reticulum, *trans*-Golgi network, and endosomes. Yet, the precise site of assembly remains unresolved. Emerging evidence suggests that membrane lipid composition may play a critical role with phosphoinositides, cholesterol, and cardiolipin, alongside membrane biophysical properties and cellular metabolic state, collectively shaping membranes where inflammasome assembles. Here, we review how lipid-dependent mechanisms regulate NLRP3 assembly across membranes and consider whether a defined membrane lipid signature governs inflammasome assembly. This understanding may have broader implications for therapeutic targeting in inflammatory and metabolic diseases.

## Abstract (web summary)

This review examines how membrane lipids, including phosphoinositides, cholesterol, and cardiolipin, along with biophysical membrane properties define organelle assembly platforms, and proposes a unified lipid model for NLRP3 recruitment.

## Introduction

Inflammasomes are large cytosolic multiprotein complexes that assemble in response to an array of microbial, environmental and endogenous danger signals, broadly referred to as pathogen-associated molecular patterns and damage-associated molecular patterns^1–4^. Recognition of these stimuli by germline-encoded pattern recognition receptors, predominantly belonging to the NOD-like receptor (NLR) family, triggers the assembly of the inflammasome complex, which initiates a cascade of events resulting in pro-inflammatory cytokine secretion and cell death^5–7^. The expression of pattern recognition receptors and associated inflammasome components is often cell-type restricted^8–11^. While inflammasomes can assemble in many cell types, including T and B lymphocytes, they are most robustly assembled in cells of the myeloid and epithelial lineages^12–14^. In particular, studies have widely utilised macrophages as model systems to identify novel inflammasome triggers, although the range of cell types being investigated is increasingly expanding to uncover additional inflammasome pathways^15–18^.

Centred around the sensor protein, the inflammasome complex consists of a cysteine protease pro-caspase-1 and the adaptor protein apoptosis-associated speck-like protein containing a CARD (ASC). Upon sensing an appropriate stimulus, the sensor protein undergoes oligomerisation. This oligomerisation provides a nucleation event that initiates the assembly of the inflammasome complex^19^. Subsequently, the adaptor protein ASC and the cysteine protease pro-caspase-1 are recruited to the NLR via the affinity of their respective PYD and CARD domains. ASC is recruited to the sensor through homotypic PYD-PYD interactions, which subsequently undergoes filamentous polymerisation. This forms a supramolecular scaffold (referred to as the ASC speck) that nucleates the recruitment of pro-caspase-1 through CARD–CARD interactions^20^. Notably, ASC is not strictly required for the assembly of all inflammasomes, although the inclusion of ASC enhances caspase-1 activation and cytokine secretion.

**Fig. 1:**
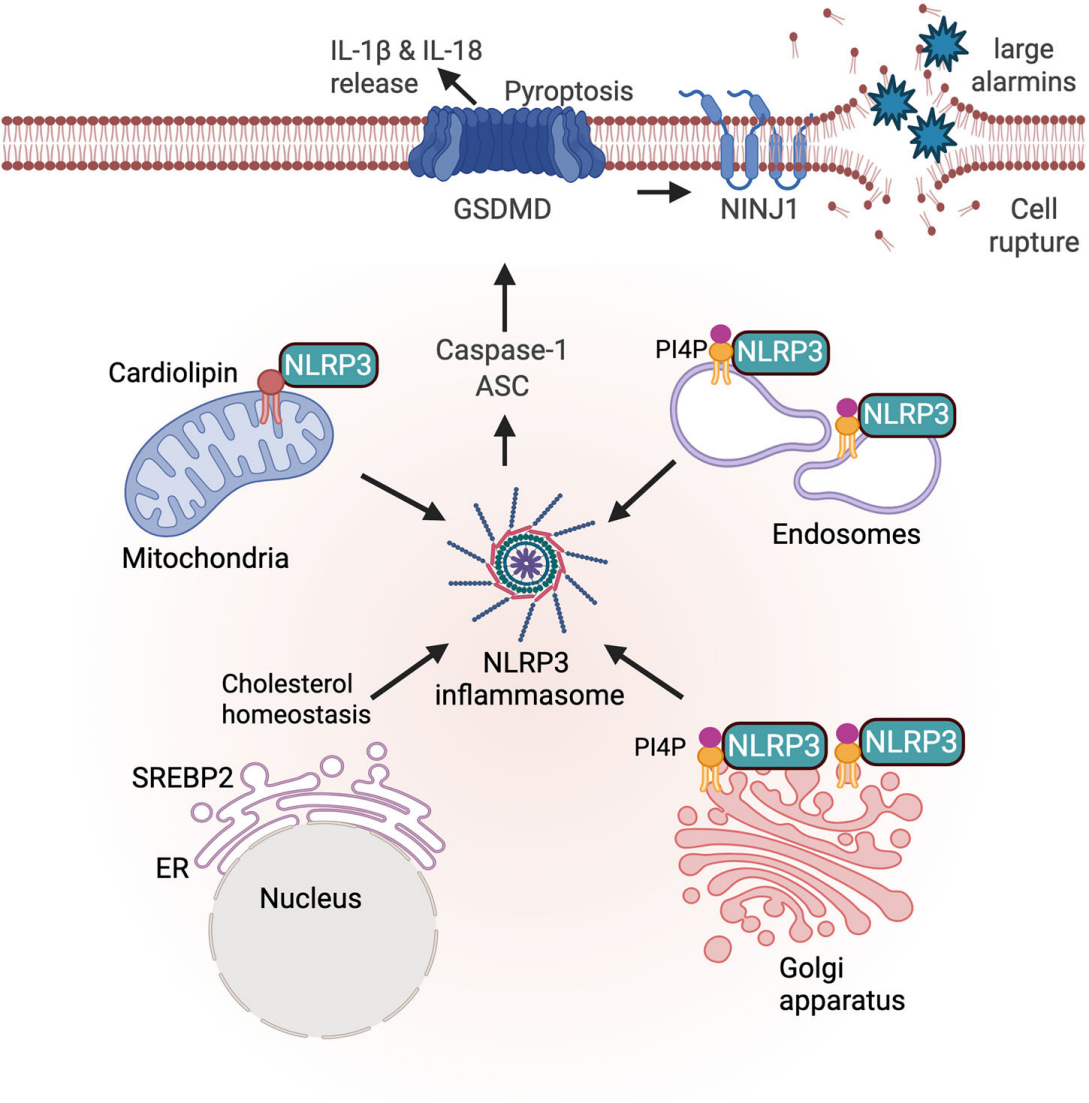
The NLRP3 inflammasome can assemble at distinct subcellular compartments. NLRP3 associates with various lipids at the indicated organelles, including cardiolipin at the mitochondria, and phosphatidylinositol 4-phosphate (PI4P) at dispersed *trans*-Golgi network (dTGN) and endosomal membranes. Additionally, cholesterol homeostasis at the endoplasmic reticulum (ER) is important, and its disruption may promote NLRP3 inflammasome activation. GSDMD Gasdermin D, NINJ1 Ninjurin-1, SREBP2 sterol regulatory element-binding protein 2. Created with BioRender.com.

Once formed, the inflammasome complex facilitates proximity-induced auto-proteolysis of caspase-1^21,22^. Active caspase-1 subsequently leads to the proteolytic cleavage of pro-inflammatory cytokines, pro-IL-1β and pro-IL-18, into their biologically active forms (Fig. 1). The mature forms of these cytokines remain within the cytosol until plasma membrane pore formation by another caspase-1-dependent substrate, Gasdermin D (GSDMD), allows their release outside the cell (Fig. 1). As such, inflammasomes not only drive the maturation but also regulate the secretion of these pro-inflammatory cytokines. GSDMD pores thus formed result in an inflammatory form of lytic cell death known as pyroptosis, in which intracellular contents are released into the extracellular space, further driving downstream pathology in many diseases (Fig. 1)^23,24^. More recent studies have shown that plasma membrane rupture during pyroptosis is executed by the membrane protein NINJ1, which facilitates the release of larger intracellular alarmins (Fig. 1)^25^.

Several inflammasomes have been identified, with each one activated in response to a distinct stimulus. However, the inflammasome formed by NLRP3 can assemble in response to a variety of triggers, including extracellular ATP acting through P2X_7_ receptor, potassium ionophores, such as nigericin, and crystalline particulates such as silica, monosodium urate and cholesterol crystals. Initially identified as a defence response to pathogens, studies have demonstrated pivotal roles of the NLRP3 inflammasome in inflammatory, metabolic and autoimmune disorders^26^. Besides, gain-of-function mutations in *NLRP3* are associated with cryopyrin-associated periodic syndromes (CAPS), a spectrum of rare autoinflammatory disorders characterised by recurrent fever and systemic inflammation^27,28^. Due to its’ clinical importance, there is a vital need to develop therapeutics targeting the NLRP3 inflammasome^26,29^. However, this necessitates understanding the disease-specific contexts and triggers that underpin aberrant NLRP3 activation. Moreover, it is known that several *NLRP3* CAPS-associated mutants are resistant to experimental NLRP3 inhibitors. Therefore, an improved understanding of fundamental mechanisms governing NLRP3 activation and inflammasome assembly is required.

Accumulating evidence in the last several years has recognised NLRP3 inflammasome assembly as a spatially regulated process, occurring at specific intracellular membranes^30,31^. However, over the years, the identity of these membrane compartments has shifted (Fig. 1). Upon exposure to activating signals, NLRP3 inflammasome has been demonstrated to assemble at the mitochondria, mitochondria-associated ER membranes, the *trans*-Golgi network (TGN), and endosomes^32–36^ (Fig. 1). Localisation of NLRP3 at these organelle membranes has been shown to be critical in facilitating its clustering and oligomerisation, and thus is required for efficient inflammasome activation. Consistent with this, structural studies support a model in which higher-order inflammasome assembly depends on membrane association^19^. The precise nature of mechanisms that underpin membrane attachment remains incompletely understood. The varied nature and composition of organelle membranes on which the inflammasome assembles suggest that NLRP3 activation is not restricted to a single membrane type. Intriguingly, forced recruitment of NLRP3 is sufficient to promote inflammasome assembly at distinct membranes^37^. Recent studies have identified key roles for lipids in NLRP3 activation^38–40^. Together, this implies that rather than a single lipid species, a defined membrane signature may be required for NLRP3 inflammasome assembly. Remarkably, pivotal roles for membrane lipids, such as phosphatidylinositol 4-phosphate (PI4P), cholesterol and cardiolipin (Fig. 1), as well as lipid modification and membrane properties, including swelling, have been identified in facilitating inflammasome assembly in recent years^34,41,42^. These observations support the notion that NLRP3 is not passively recruited to organelles but is instead guided by a defined membrane identity, which may comprise of a combination of lipid composition and biophysical properties. In this Review, we discuss the current evidence and propose lipid composition, lipid-dependent modification and membrane properties as central in regulating the spatial assembly of the NLRP3 inflammasome.

## Specific phosphoinositides as determinants of NLRP3 inflammasome assembly

### Golgi PI4P and NLRP3 inflammasome assembly

**Fig. 2:**
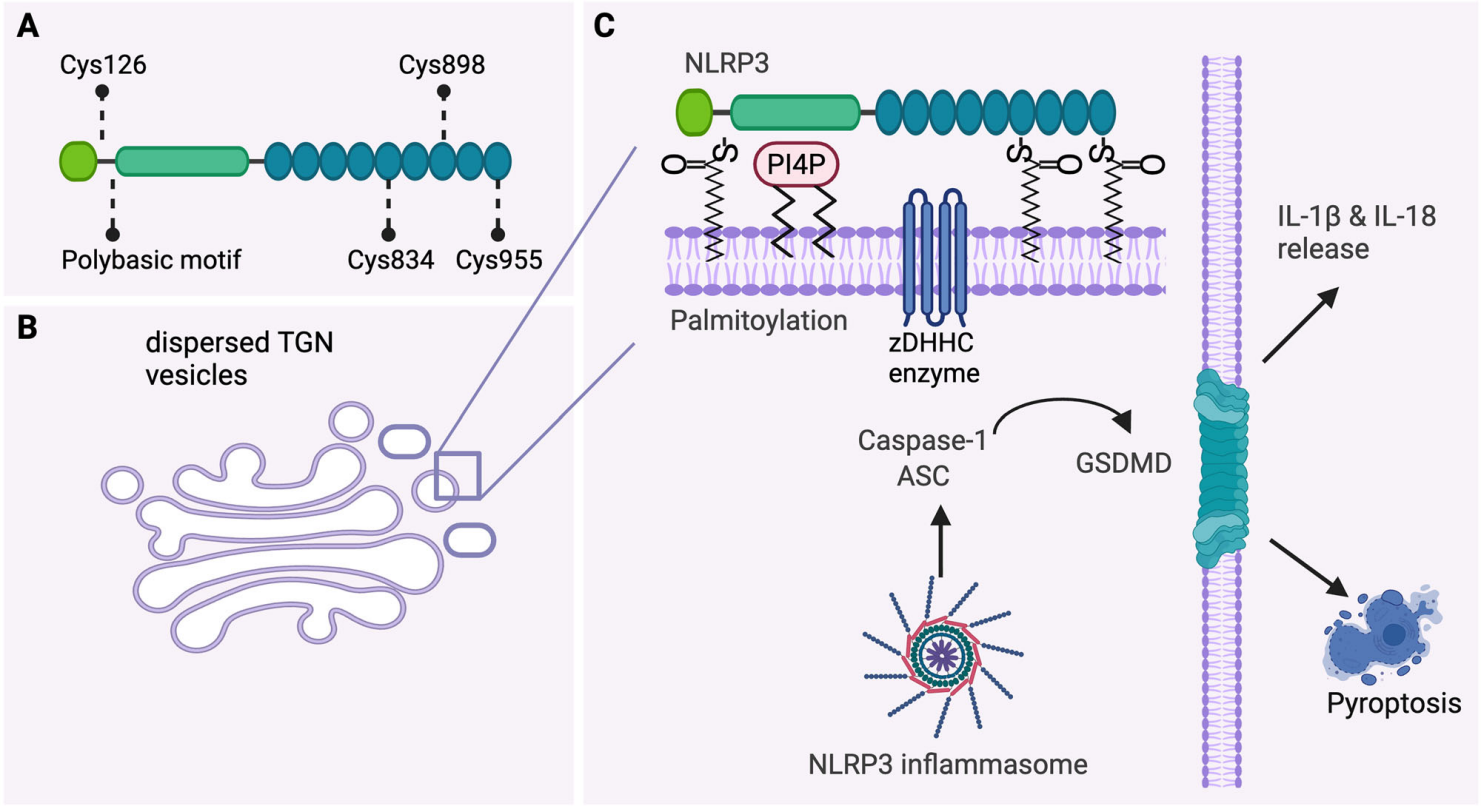
NLRP3 palmitoylation and polybasic residue enable NLRP3 dispersed *trans*-Golgi network (dTGN) localisation. **A** NLRP3 domain structure depicting the polybasic motif and NLRP3 palmitoylation sites. **B** NLRP3 activation results in TGN dispersion, at which phosphatidylinositol 4-phosphate (PI4P) accumulates, leading to NLRP3 recruitment. **C** NLRP3 association with the TGN is further stabilised by palmitoylation. This results in NLRP3 inflammasome activation, resulting in the formation of Gasdermin D (GSDMD) pores and release of cytokines IL-1β and IL-18. zDHHC zinc finger DHHC domain-containing. Created with BioRender.com.

A consensus on the organelle identity where the NLRP3 inflammasome assembles has evolved over time. Specifically, recent work has identified TGN membranes as a central platform for NLRP3 recruitment and activation^34^. A key role for a conserved polybasic motif consisting of four lysine residues (K127–130 in human NLRP3) has been identified^34^ (Fig. 2A). Located in the linker region between the NLRP3 PYD and NACHT domains, the positively charged region interacts with the TGN membrane-localised acidic phosphoinositide PI4P through electrostatic interactions (Fig. 2B, C). In this context, these PI4P-rich membranes provide a platform for NLRP3 clustering into puncta, which nucleates ASC polymerisation and initiates downstream inflammasome signalling. A recent report proposed that ceramides may also contribute to NLRP3 recruitment at the TGN (Table 1)^43^. Intriguingly, only a small fraction of the constitutively expressed NLRP3 undergoes activation-dependent oligomerisation. Yet, this is sufficient to nucleate the formation of a large ASC speck at TGN membranes. Consistent with this, NLRP3 membrane association lowers the threshold for NLRP3 oligomerisation. Accordingly, CAPS-associated NLRP3 mutants form dispersed TGN (dTGN)-localised puncta even in the absence of stimulation^34^. Importantly, NLRP3 interaction with dTGN membranes is not limited to K127–130 residues. Mutations in several additional positively charged residues within the polybasic region (K134, R137, R138 and R141) also impaired NLRP3 oligomerisation^34^, suggesting that an array of basic motifs may be necessary to mediate stable electrostatic interactions with PI4P-enriched membranes.

**Table 1:** Function of lipids and related metabolites in NLRP3 localisation and activation

Lipids and related metabolites	Function in NLRP3 localisation and/or activation	Key references
PI4P	PI4P recruits NLRP3 to the dispersed TGN, providing a scaffold for NLRP3 clustering.	Chen and Chen^34^
Cholesterol	Cholesterol levels in the ER and SREBP2-mediated cholesterol biosynthesis are critical to inflammasome assembly.	de la Roche et al.^42^, Guo et al.^61^
Ceramides	Ceramide-enriched vesicles serve as a platform for NLRP3 recruitment and subsequent inflammasome assembly.	Liu et al.^43^
Cardiolipin	NLRP3 binds to mitochondrial cardiolipin externalised upon mitochondrial dysfunction.	Iyer et al.^41^
Cholesterol crystals	Lysosomal damage by cholesterol crystals activates the NLRP3 inflammasome.	Duewell et al.^91^
Saturated fatty acids	Saturated fatty acids, including palmitic and stearic acid, activate the NLRP3 inflammasome through free fatty acid crystallisation and lysosomal damage.	Wen et al.^94^, Karasawa et al.^95^, Gianfrancesco et al.^96^
Unsaturated fatty acids	Eicosapentaenoic acid (EPA) and docosahexaenoic acid (DHA) omega-3 fatty acids inhibit HFD-induced NLRP3-mediated metabolic disorders in mice.	Yan et al.^97^, Karasawa et al.^95^, Gianfrancesco et al.^96^
Oxidised LDL	Uptake of oxidised LDL via CD36 receptor promotes lysosomal crystal formation and NLRP3 inflammasome activation.	Sheedy et al.^98^
Palmitic acid	FASN-driven NLRP3-lipidation by palmitic acid promotes NLRP3 translocation to dispersed TGN vesicles.	Leishman et al.^77^

Phosphoinositides are key regulators of membrane identity, participating in numerous signalling and trafficking processes within the cell^44^. A role for PI fatty acid chains has been demonstrated in NLRP3 inflammasome priming and activation^45^. Given that NLRP3 is associated with multiple membrane compartments, and that phosphorylated phosphoinositide species are distributed across cellular membranes^46^, it is plausible that NLRP3 may interact with additional phosphoinositides beyond PI4P. Intriguingly, in protein-lipid overlay assays, a purified FLAG-tagged NLRP3 fragment spanning residues 127–146 bound to several additional negatively charged phospholipids, including PI(3,5)P2, PI(4,5)P2, PI3P and phosphatidic acid^34^. The physiological significance of binding to most of these phosphoinositides remains to be established. Nevertheless, these observations raise the possibility that differences in binding affinity or local lipid stoichiometry may modulate NLRP3 recruitment to varied cellular compartments rich in diverse phosphoinositides. In addition, PI4P is enriched not only at the Golgi apparatus but is also present at the plasma membrane (and endosomal membranes)^47,48^. Notably, experimental evidence points that the total size of the PI4P pool at the plasma membrane may be up to 3-fold higher than that at the Golgi^48^. Yet, inflammasome nucleation is not typically observed at the plasma membrane, indicating that additional factors beyond PI4P availability may contribute to the selective NLRP3 recruitment to specific membrane compartments.

Contrary to the above, it has been very recently shown that membrane localisation does not directly govern inflammasome activation. By employing NLRP3 constructs that were targeted to distinct subcellular compartments, the authors found that NLRP3 clustering at membranes was sufficient to promote inflammasome activation^37^. However, membrane-targeted NLRP3 variants that lacked the polybasic phospholipid-binding motif still retained the ability to form puncta^37^. Moreover, protein scaffolds such as the aggregation-prone TDP43 could substitute for membrane association and induce NLRP3 clustering and inflammasome assembly. These findings indicate that PI4P-dependent recruitment to TGN and endosomes or NLRP3 membrane association is not strictly required. Nevertheless, these studies raise several additional questions. For instance, is the polybasic motif alone sufficient for NLRP3 membrane recruitment and localisation, or are additional membrane features required? Do other immune receptors with polybasic regions also localise to the TGN? Indeed, Golgi PI4P pool and the PI4P-associated kinase PI4KB have been shown to regulate the cytosolic DNA sensing cGAS-STING pathway, implying broader roles for PI4P availability in influencing innate immune signalling^49,50^.

### Endosomal PI4P and NLRP3 recruitment and activation

**Fig. 3:**
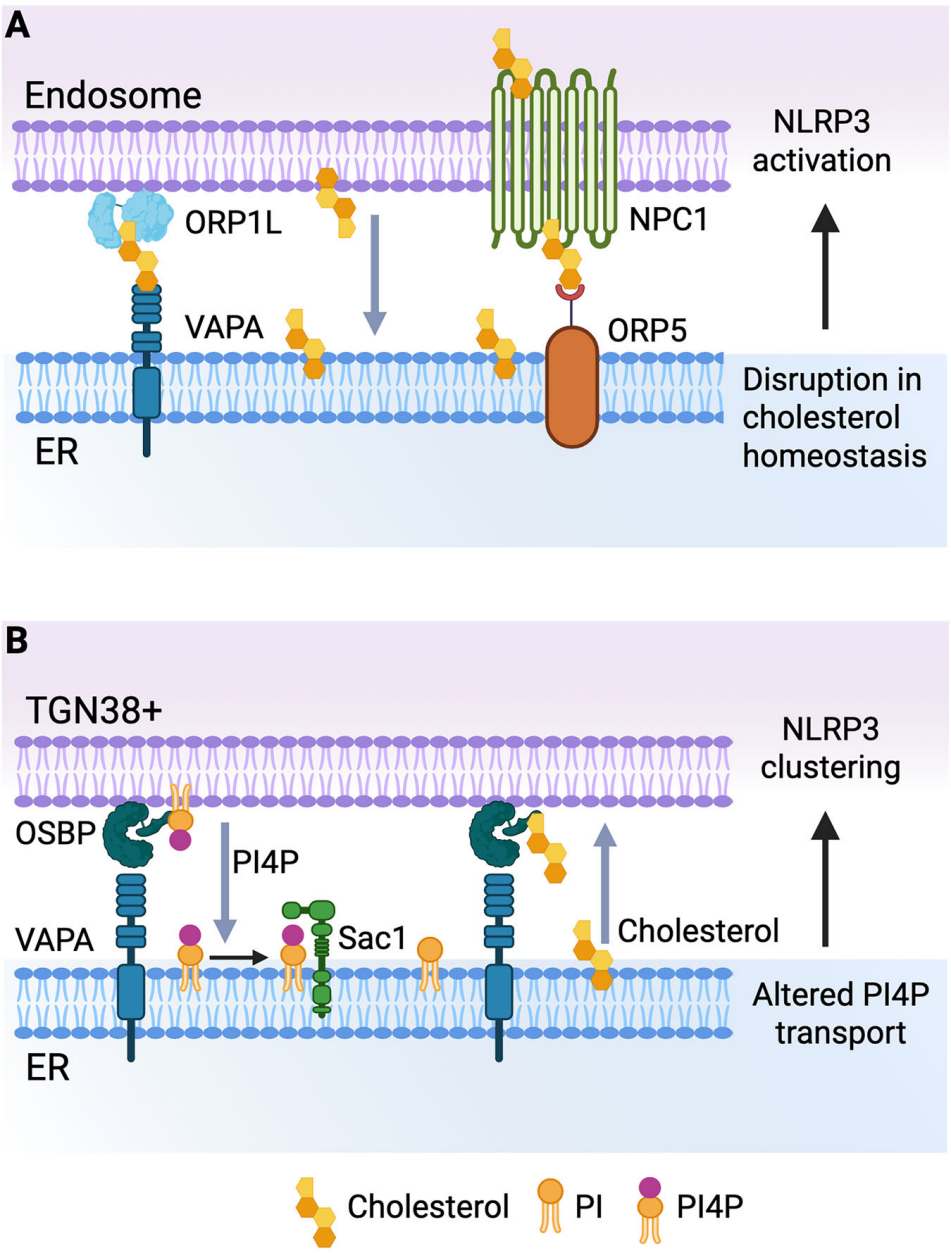
Cholesterol and PI4P transport in NLRP3 inflammasome assembly. **A** Cholesterol transport from endosomes to the endoplasmic reticulum (ER) can be mediated by NPC1 and ORP5 contact sites, or by interactions formed by ORP1L and VAPA. Disruption of ER cholesterol homeostasis leads to NLRP3 inflammasome assembly. **B** Reciprocal PI4P–cholesterol transport at ER-TGN contact sites shapes membrane composition and promotes NLRP3 inflammasome assembly. PI4P at the TGN is returned to the ER, where it is degraded by the phosphatase Sac1. In return, cholesterol is transported from the ER to the TGN. Altered PI4P transport or accumulation at the TGN and endosomal membranes enables NLRP3 clustering and activation. PI4P phosphatidylinositol 4-phosphate, TGN *trans*-Golgi network, OSBP oxysterol-binding protein, ORP1L oxysterol-binding protein-related protein 1 long, VAPA VAMP-associated protein A, NPC1 Niemann-Pick C1. Created with BioRender.com.

More recently, the PI4P-positive TGN compartment where the NLRP3 inflammasome assembles has been redefined as endosomal in nature^35,36^. Although endosomal protein EEA1 was observed in the same PI4P-positive compartment in the initial study, it was proposed to be missorted to Golgi-derived TGN46-positive vesicles^34^. New studies demonstrate that NLRP3 activating stimuli cause disruption of ER-endosome membrane contact sites (EECS) and consequently a defect in retrograde transport from the endosomal-to-*trans*-Golgi network (ETT)^36^. During homeostasis, EECS and retrograde trafficking is required for the transfer of PI4P from endosomes to the ER, where PI4P is hydrolysed by the phosphatase Sac1, which maintains the PI4P gradient across membranes (Fig. 3A, B). Abolition of EECS or ETT impairs this PI4P turnover, resulting in endosomal accumulation of TGN46 (TGN38 in rodents), which cycles between PM, endosomes and TGN^51,52^.

Retrograde transport from endosomes to the Golgi recycles essential membrane proteins and lipids back to the Golgi apparatus, maintaining cellular homeostasis. In mouse bone marrow-derived macrophages, disruption of retrograde trafficking alone by monensin was insufficient to activate the inflammasome^35^. Instead, this disruption must accompany NLRP3 activating stimuli, indicating that perturbed trafficking and PI4P accumulation is necessary but not sufficient for inflammasome activation. Additional events, including changes in membrane composition or post-translational modifications, may be required. On the other hand, knock-out of ER-localised VAPA/B in HeLa cells, which disrupts EECS, resulted in constitutive NLRP3-EGFP recruitment to PI4P-enriched endosomes^36^ (Fig. 3A). Remarkably, this could occur in the absence of inflammasome activators, suggesting that NLRP3 overexpression may lower the threshold for membrane recruitment and override upstream regulatory steps, such as LPS priming and post-translational control^36^. Consistently, disruption of EECS in THP-1 cells resulted in inflammasome activation and pyroptosis by LPS alone.

The primary endosome-associated PI is PI3P. Consistent with this, the NLRP3 assembly compartment was found to be positive for TGN38, PI4P and PI3P^36^, implying that NLRP3 is recruited to a compartment with mixed phosphoinositide identity^53,54^. While protein-lipid overlay assays demonstrated NLRP3 affinity for PI3P^36^, whether PI3P directly contributes to endosomal NLRP3 recruitment and which NLRP3 residues mediate this association remains unexamined. Moreover, direct interaction between PI4P and NLRP3 on endosomes remains to be determined, as forced binding of NLRP3 with PI4P was insufficient to induce inflammasome assembly. This may indicate that PI4P-mediated recruitment, while necessary, is not sufficient to activate inflammasome assembly in the absence of additional signals. While ETT contributes to endosomal PI4P accumulation, it is possible that NLRP3 also senses other lipids or proteins that accumulate in endosomes in response to impaired TGN cargo transport. This may generate a membrane environment that is permissive for NLRP3 recruitment and nucleation. As such, PI4P accumulation may be necessary rather than the sole determinant of NLRP3 recruitment. Overall, these findings support a model in which NLRP3 recruitment is not driven by a single lipid species but instead integrates multiple features of membrane identity, including phosphoinositide composition, to promote NLRP3 spatial assembly.

## Cholesterol and membrane lipid composition in NLRP3 inflammasome assembly

Lipid composition is fundamental to membrane function, facilitating structural integrity and fluidity, which together help shape key cellular processes such as signalling and transport^55^. Specific lipid types (including phospholipids, sphingolipids and sterols) and their relative levels maintain membranes, allowing the formation of specialised domains that enable the above functions. As such, variations in lipid composition can result in distinct membrane environments influencing protein recruitment and signalling responses^56,57^. The primary site for lipid synthesis and exchange, the ER forms membrane contact sites with other organelles, through which lipids such as cholesterol, as well as calcium and other signalling molecules, are transferred^58^. While maintaining only low levels of total cholesterol content, ER sterol levels (1–5%) have been shown to play an important role in regulating the NLRP3 inflammasome^42^. Consequently, blocking cholesterol export from endolysosomal compartments, leading to reduced ER cholesterol levels, results in reduced inflammasome activation (Fig. 3B and Table 1). In agreement, acute depletion of ER sterols using statins produced a similar effect, with reduced caspase-1 activation, IL-1β secretion and pyroptosis ^42^.

The exchange of cholesterol between endosomes and the ER is predominantly mediated at membrane contact sites^59^. This involves the endosomal membrane protein NPC1, which transfers cholesterol to ORP5 for delivery to the ER (Fig. 3A). Alternatively, cholesterol can be transported by ORP1L, which binds the small GTPase Rab7 and Rab7-interacting lysosomal protein on endosomes^59^. Under limiting cholesterol conditions, the FFAT motif of ORP1L interacts with VAP proteins on the ER, allowing tighter ER–endosome contact^60^ (Fig. 3A). Whether cholesterol transfer through these sites contributes to inflammasome activation remains to be determined. However, NPC1 deficiency (*Npc1*^*−/−*^) in macrophages has been associated with reduced ER cholesterol levels and impaired inflammasome activation^42^. In agreement with this, knockout of oxysterol-binding protein (OSBP), which transfers cholesterol from the ER to the Golgi (coupled to reciprocal PI4P transport), enhanced inflammasome activation^36^ (Fig. 3B). Altogether, this implies that perturbations in ER cholesterol homeostasis can influence NLRP3 assembly. By contrast, other studies observed that reduced ER sterol levels may lead to NLRP3 recruitment to the Golgi, potentially contributing to inflammasome activation^61^. Overall, these findings support the notion that cholesterol flux between organelles, rather than steady-state levels alone, may contribute to defining membrane environments that regulate NLRP3 assembly. Moreover, these studies collectively propose a role for ER cholesterol homeostasis in modulating NLRP3 inflammasome assembly.

Besides the ER, mitochondrial membrane lipid composition also contributes to inflammasome assembly. Specifically, it has been demonstrated that NLRP3 binds to mitochondria through interaction with the acidic phospholipid cardiolipin^41^ (Fig. 1 and Table 1). Although cardiolipin is exclusively localised to the inner mitochondrial membrane, where it accounts for ~10–20% of total phospholipid content^62^, mitochondrial stress can lead to its externalisation to the outer mitochondrial membrane, where it interacts with cytosolic NLRP3. By employing linezolid, an antibiotic used to treat serious Gram-positive infections^63^, but prolonged use is associated with myelosuppression and mitochondrial toxicity^64^, it was demonstrated that drug-induced mitochondrial stress promotes cardiolipin translocation to the outer mitochondrial membrane^41^. Notably, the requirement for cardiolipin is not limited to linezolid, which facilitates the ROS-independent NLRP3 activation pathway. Canonical NLRP3 stimuli, such as ATP, which activate NLRP3 in a ROS-dependent manner, also converged upon cardiolipin externalisation^41^, indicating a shared lipid-dependent mechanism that links mitochondria to inflammasome activation.

NLRP3 recruitment to mitochondria supports inflammasome assembly at this compartment. Accordingly, in a broken cell assay using LPS-primed mouse macrophages, the addition of cardiolipin (but not phosphatidylcholine, phosphatidylserine or phosphatidic acid) triggered caspase-1 activation in a dose-dependent manner^41^. Moreover, cell lysates from mouse macrophages expressing HA-tagged NLRP3 showed strong binding to cardiolipin in protein–lipid overlay assays. Additionally, weaker interactions were observed for phosphatidic acid and 3-sulfogalactosylceramide. Intriguingly, this interaction extended to several phosphoinositides when purified His-tagged human NLRP3 was used in overlay assays^41^. Like phosphoinositides, cardiolipin is an acidic (negatively charged) phospholipid. Consequently, it is tempting to speculate that NLRP3 recruitment to mitochondrial cardiolipin occurs via the same polybasic motif that mediates PI4P binding (linker region between PYD and NACHT domains). By contrast, cardiolipin interacted with FLAG-tagged NLRP3 via the LRR domain^41^, indicating that distinct NLRP3 regions can interact with different lipid species.

Other studies have shown the involvement of additional factors in NLRP3 mitochondrial recruitment. NLRP3 stimuli have been shown to activate mitochondria-associated glycogen synthase kinase 3β (GSK3β), which directly binds to NLRP3, facilitating its transient association with mitochondria before subsequent redistribution to the TGN^65^. Here, activation of GSK3β has been proposed to regulate phosphatidylinositol 4-kinase 2A (PI4K2A), a Golgi- and endosome-resident enzyme that catalyzes PI4P synthesis^65,66^. While mitochondrial recruitment of NLRP3 is supported by several studies^67,68^, more recent work has failed to observe NLRP3 association with mitochondria in experiments performed in *Asc*-deficient macrophages^34^. This may be because of differences in experimental conditions or that stable NLRP3 localisation is led by ASC-dependent assembly. This may additionally imply that mitochondrial association is a transient step in inflammasome localisation. Nonetheless, these studies promote the idea that NLRP3 may transiently sample multiple membrane compartments before recruitment to PI4P-enriched TGN or endosomal membranes for inflammasome assembly.

## Membrane properties governing NLRP3 inflammasome assembly

While the above studies support roles for specific lipids in NLRP3 recruitment, broader membrane properties have also been suggested to contribute to inflammasome assembly. The physical state of the membrane compartment, including luminal acidity, osmotic swelling, surface tension and the ionic environment are important to NLRP3 recruitment and oligomerisation. Consequently, membranes additionally provide a biophysical context that defines an inflammasome assembly platform. It has been demonstrated that NLRP3 is selectively recruited to endocytic compartments that undergo vacuolar ATPase-dependent osmotic swelling^69^. Intriguingly, similar to recent evidence with canonical NLRP3 stimuli, osmotic swelling resulted in mCherry-NLRP3 recruitment to LAMP1-, PI4P- and PI(3,5)P2-positive compartments. Notably, NLRP3 is preferably recruited to LAMP1-positive late-endosomes over the early endosome compartment in HeLa cells. The nature of the NLRP3 compartment in HeLa cells remains ambiguous, considering that PI4P and PI(3,5)P2 usually occupy distinct compartments^48^. Remarkably, experiments that reduced hydrostatic pressure on these vesicles abolished NLRP3 recruitment and IL-1β release, implying that membrane tension is necessary for NLRP3 inflammasome activation. Consistent with this, a recent study reported fragmented and curved LPS membranes as stronger inducers of the caspase-4 non-canonical inflammasome^70^. Taken together, these findings indicate that v-ATPase activity, vesicle swelling and membrane tension are part of a single pathway that leads to inflammasome activation.

The ionic pathway has been further explored in a study that examined the role of volume-regulated anion channel (VRAC). Using LRRC8A knock-out macrophages, it was demonstrated that chloride efflux through VRAC is required for hypotonicity-induced NLRP3 activation but not for inflammasome activation by classical DAMPs^71^. This suggests that osmotic stress engages a pathway involving cell swelling, VRAC activation, chloride efflux and subsequent potassium loss. This has been further corroborated by a study that demonstrated efflux of taurine, a major intracellular osmolyte, through VRAC^72^. Depletion of taurine resulted in impaired Na^+^/K^+^-ATPase activity. Na^+^/K^+^-ATPase pump is critical in maintaining the ionic gradient required for potassium efflux. Consequently, taurine loss through the pump amplifies ionic imbalance upstream of NLRP3 activation. In agreement, blocking VRAC or replenishing taurine resulted in restored ionic balance and IL-1β release^72^. Collectively, these observations propose that the physical properties of the membrane may additionally contribute alongside lipid composition to regulate NLRP3 recruitment and activity. Importantly, these observations do not favour a specific compartment as any of the subcellular compartments could acquire these properties to support inflammasome assembly. In this context, specific lipid composition and the physical state of that membrane likely determine whether NLRP3 activation actually proceeds.

## NLRP3 palmitoylation and inflammasome assembly

Several post-translational modifications, including phosphorylation, ubiquitination and SUMOylation, have been shown to modulate inflammasome assembly. Intriguingly, a lipid-mediated post-translational modification, palmitoylation, has recently been demonstrated to further calibrate NLRP3 interactions with membrane environments. Palmitoylation involves the reversible attachment of a 16-carbon fatty acid palmitate to specific (predominantly cysteine) residues of target proteins^73^. The reaction is carried out by enzymes known as palmitoyl acyltransferases, a family of enzymes characterised by a conserved zinc finger domain containing Asp-His-His-Cys (DHHC) tetrapeptide motif in their catalytic site^74^ (Fig. 2C). NLRP3 is palmitoylated at multiple cysteine residues by diverse zDHHC enzymes, with each modified site contributing to distinct stages of the inflammasome activation process. Consequently, the outcome of NLRP3 palmitoylation depends very much on the site and the stage of inflammasome activation.

The precise cellular localisation of NLRP3 in the resting stage and upon activation remains debatable. NLRP3 localises both in the cytoplasm and on membranes prior to activation^19,75^. Upon stimulation, NLRP3 may traffic to the mitochondria, ER, dTGN and endosomes before proceeding to the MTOC for assembly. Notably, NLRP3 membrane localisation has been shown to enhance inflammasome activation^19,75^. A characteristic function of palmitoylation, NLRP3 modification at cysteine residues 126, 898 and 955 enables NLRP3 recruitment to dTGN and endosome membranes, thereby aiding inflammasome activation^75–77^ (Fig. 2A and Table 2). Cys898 modification, which occurs upon LPS priming, has been directly linked to de novo fatty acid synthesis by fatty acid synthase^77^. Consequently, inhibition of fatty acid synthase impaired NLRP3 palmitoylation and localisation to dTGN membranes, thereby highlighting a key role for endogenous lipid synthesis in inflammasome activation^77^. Moreover, these findings directly link cellular lipid metabolism to NLRP3 inflammasome spatial regulation. This is in agreement with previous studies linking metabolic flux to NLRP3 activation^78^. However, palmitoylation alone is unlikely to be the only membrane anchor for NLRP3. NLRP3 palmitoylation must first precede by transient interaction of NLRP3 with PI4P-rich TGN compartment^34^. In agreement, disruption of NLRP3-PI4P association by mutating the NLRP3 polybasic region abolished Cys126 palmitoylation, impaired NLRP3 membrane association, and resulted in the formation of inactive oligomeric structures^19,76^. In contrast to the above research, other studies observed a role for Cys126 only at the activation step when NLRP3 transitions from TGN to dTGN^75^. Additionally, varied zDHHC enzymes have been reported for Cys126 palmitoylation with both ER-localised zDHHC1 and Golgi-localised zDHHC7 governing palmitoylation at this site^75,76^.

**Table 2:** Key function of distinct palmitoylation sites in NLRP3 inflammasome activation

Mouse NLRP3 residue	Human NLRP3 residue	Functional role of palmitoylation	Reference
Cys126	Cys130	NLRP3 localisation to dispersed TGN vesicles	Yu et al.^76^, Williams and Peden^99^
Cys419	Cys419	Facilitates NLRP3-NEK7 interaction	Hu et al.^75^
Cys 834/835	Cys837/838	Facilitates NLRP3-NEK7 interaction	Zheng et al.^79^
Cys841	Cys844	NLRP3 degradation by autophagy	Wang et al.^80^
Cys898	Cys901	NLRP3 localisation to dispersed TGN vesicles	Leishman et al.^77^
Cys126, Cys955	Cys130, Cys958	NLRP3 trafficking between mitochondria, TGN and endosomes	Nie et al.^75^

NLRP3 palmitoylation and subsequent phosphorylation at distinct cysteine and serine residues, respectively, regulate the sensor protein interaction with MTOC-localised NEK7^75^. During membrane transport, NLRP3 is sequestered in an inactive ‘cage’ structure in which the LRR domain of NLRP3 blocks access to NEK7, preventing ASC recruitment. Sequential NLRP3 palmitoylation first at Cys958 (murine Cys955) followed by Cys130 (murine Cys126) during priming and activation, respectively, directs NLRP3 to specific membranes before proceeding to MTOC^75^. Here, NLRP3 is phosphorylated at Ser265 by LATS1/2, which relieves autoinhibition, permitting NEK7 interaction (Table 2)^75^. As such, NLRP3 palmitoylation coordinates with other post-translational modifications to regulate NLRP3 subcellular trafficking and activation. Remarkably, NLRP3 palmitoylation here was found to increase only during nigericin stimulation, indicating a role for Cys126 during later stages of NLRP3 activation.

Beyond membrane association, NLRP3 palmitoylation may have additional roles. Modification at Cys834/835 does not affect NLRP3 localisation to the TGN^79^. Instead, Cys834/835 palmitoylation modulates NLRP3 oligomerisation, facilitating NEK7 interaction and formation of ASC aggregates (Table 2)^79^. Moreover, zDHHC12-mediated NLRP3 palmitoylation at Cys844 (Cys841 in mouse) has been shown to inhibit inflammasome activation by autophagy^80^. Contrarily, other studies found no reduction in NLRP3 palmitoylation in either zDHHC12-knockdown cells or in cells where NLRP3 was mutated at Cys843/844 residues^75,79^. Still other studies found that palmitoylation promotes NLRP3 phase separation, which is required for NLRP3 activation^81^. Notably, both the NLRP3 polybasic region and localisation to the TGN46 compartment were found dispensable for inflammasome activation^81^. Nonetheless, this implies that palmitoylation can have distinct, sometimes opposing effects, depending on the modified site

## Lipid-dependent spatial organisation in innate immune signalling

Emerging evidence points out that lipid-dependent spatial organisation may be a broader principle in innate immune signalling. Studies have described key roles for membrane lipid composition and lipid environment in the activation of the DNA-sensing STING pathway. Modulation of PI4P levels at the TGN directly calibrates STING-mediated interferon signalling, while STING variants unable to bind PI4P fail to traffic from the ER to the Golgi and generate interferon and cytokine production^50^. The requirement for PI4P is validated by another study, though the latter study highlighted PI4P role in STING transport from the Golgi to the endosome^49^. STING transport from the ER to the Golgi additionally requires palmitoylation at the Cys88/91 residues^82^. The lipid modification enables STING to recruit TBK1, leading to IRF3 phosphorylation and type I interferon production. In agreement, inhibition of palmitoylation blocked STING activation^82^. This phenomenon could be replicated in STING variants observed in patients with early-onset systemic inflammation, which exhibit a gain-of-function phenotype and stimulate interferon independently of cGAMP. Inhibition of palmitoylation or mutation of Cys88/91 to serine residues, which prevents palmitoylation, blocked interferon responses^82^. These studies may lead to the development of new treatments for cytosolic DNA-triggered autoinflammatory disease.

Lipid-dependent membrane engagement also determines other inflammatory cell death pathways. During necroptosis, the activated mixed lineage kinase-domain-like (MLKL) protein undergoes oligomerisation dependent on positively charged amino acids in the N-terminal four-helical bundle domain of MLKL^83^. The positively charged domain binds to PIPs, allowing MLKL recruitment to the plasma membrane and subsequent membrane disruption during necroptosis. By contrast, mutants lacking these positively charged residues failed to localise to the plasma membrane and significantly reduced necroptotic cell death^83^. Further to NLRP3 and MLKL, studies on GSDMD revealed that the protein interacts with lipids to cause pyroptotic membrane pore formation^23,84^. The N-terminal fragment of GSDMD associates with acidic phospholipids, including PIPs and cardiolipin, before oligomerising to form membrane pores in mammalian cells and artificially transformed bacteria. In addition, GSDMD has been shown to undergo palmitoylation at Cys191 (Cys192 in mouse), which is required for efficient membrane translocation of the GSDMD N-terminal fragment and subsequent pore formation^85–87^. These observations imply a broader role for membrane lipid composition and spatially organised lipid microenvironments in innate immune signalling.

## Metabolic disease and lipid-dependent regulation of inflammasome signalling

Increasing evidence indicates that disease-associated lipid alterations can directly influence NLRP3 inflammasome activation. Metabolic diseases such as obesity and atherosclerosis are associated with inflammasome activation^88–90^. Earlier studies indicated a role for cholesterol crystals in promoting atherosclerosis in mouse models of disease (Table 1)^91^. Similarly, obesity-associated inflammation has been linked to aberrant NLRP3 activation in adipose tissue macrophages^92^, suggesting that chronic metabolic stress promotes inflammasome signalling in vivo.

Beyond inflammatory signalling, altered lipid environments may directly influence the spatial organisation and assembly of the NLRP3 inflammasome. Intracellular cholesterol trafficking and biosynthesis involving ER cholesterol levels and SREBP2-mediated NLRP3 translocation to the Golgi have been shown to impact inflammasome assembly and activation^42,61^. Similarly, a high-fat diet is a precursor to insulin resistance, obesity and type 2 diabetes. As such, fatty acid biosynthesis has also been shown to directly facilitate inflammasome assembly while genetic deletion of NLRP3 improved metabolic outcomes^77,89^. Together, these studies support a model in which pathological lipid remodelling actively reshapes membrane lipid environments that determine inflammasome assembly and activation.

## Conclusions and future perspectives

The spatial localisation of the NLRP3 inflammasome remains context-dependent and has evolved over time. During inflammasome assembly, NLRP3 may transition through multiple subcellular compartments before the complex fully assembles. Each of these transitions is tightly controlled and involves the coordinated action of several processes, including lipids and membrane properties, that collectively define the inflammasome assembly site. Considering that published literature demonstrates that varied compartments can support inflammasome localisation, this implies that NLRP3 assembly does not require a specific organelle but rather a membrane environment that is permissive for assembly. In recent years, membrane lipids have been demonstrated to influence this site. Therefore, we propose that a minimal membrane signature consisting of membrane lipid composition, membrane properties, compartment positioning and ionic context is critical in defining inflammasome localisation. Therefore, no single lipid is sufficient, but rather NLRP3 integrates multiple cues to define its site of assembly. For instance, endosome-ER cholesterol efflux, PI4P gradients across membranes, and fatty acid synthesis serve to provide key substrates that shape membrane environments. In particular, recent studies have shown that NLRP3 palmitoylation underpins NLRP3 complex formation. However, given that different cysteine sites influence different outcomes, this modification may tune NLRP3 interaction with cellular membranes rather than dictating localisation per se. Beyond changes in lipid abundance and distribution, it is critical to determine how lipid oxidation influences inflammasome assembly and localisation. Ferroptosis-associated lipid peroxidation significantly alters membrane properties, thus raising the possibility that oxidised phospholipids may directly modulate inflammasome assembly and signalling ability^93^. Determining how membrane lipid oxidation reshapes innate immune signalling may be an important future direction linking ferroptosis, membrane biology and inflammasome regulation.

A more comprehensive approach examining inflammasome assembly in relation to other subcellular compartments is required to fully address inflammasome localisation. This may include using systems that allow precise control of membrane composition. This understanding is key for disease and therapeutic targeting, as membrane lipid composition and metabolic state may lower the threshold for NLRP3 clustering and oligomerisation. Consequently, targeting membrane properties and lipid pathways may provide better therapeutic options in diseases such as CAPS. Therefore, a minimal membrane signature required for inflammasome assembly is likely to be a composite of both qualitative and quantitative thresholds. Can different compartments achieve similar permissive states for inflammasome localisation? It is likely that there are also cell-type-specific differences in the membrane signature required, and consequently, where the inflammasome assembles. Understanding how lipid composition within membranes defines NLRP3 integration of activating signals may explain inflammasome activation across diverse stimuli and cellular compartments.

## Supplementary information

Transparent Peer Review File
