# Supplementary material for: Spatial control of NLRP3 inflammasome assembly by membrane lipid composition
Source: Commun Biol. 2026 Jun 23;9:857. doi: 10.1038/s42003-026-10541-0 (PMC13291237; doi:10.1038/s42003-026-10541-0)
Supplement: Supplementary file 1 — Transparent Peer Review File [file 42003_2026_10541_MOESM1_ESM.pdf]

nature portfolio

Peer Review File

Spatial control of NLRP3 inflammasome assembly by membrane lipid composition

Corresponding Author: Dr Paras Anand

This file contains all reviewer reports in order by version, followed by all author rebuttals in order by version.

Version 0:

Reviewer comments:

Reviewer #1

(Remarks to the Author)
In this excellent mini-review article, Anand and Aljadeed provide a thoughtful and timely overview of the emerging literature demonstrating that lipid composition, especially at distinct organelles, can profoundly shape NLRP3 inflammasome assembly and signaling. They do a magnificent job of describing relevant molecular details that govern this crosstalk between lipid biology and the NLRP3 inflammasome. In addition, they nicely highlight the significance of this line of work for the design of improved NLRP3 inflammasome-directed therapies. The authors do an especially impressive job of covering recent discoveries in this field, including highlighting high-impact work that was just recently published. They cover the major subject areas in a fair and balanced manner. The figures are very attractive and professionally done, and will serve as a great resource for others in the field. Overall, this is an excellent review article that is extremely well written and organized. Below, I list a few very minor critiques, that once addressed, should help to make this already outstanding review article even better.
Specific comments:
1. The acronyms ER and TGN should probably be defined in the abstract.
2. It appears that a space is needed between “nucleates” and “ASC” in line 133.
3. They should double-check the citation style in line 175, as it appears to be inconsistent with the numbered approach used throughout the rest of the mini-review article.
4. SREBP2 overlaps with the ER structure in Figure 1, which makes it hard to read. They should consider moving it to improve readability.
5. To increase the scope of the mini-review article, it would be interesting to know how lipid oxidization as occurs in ferroptosis, influences NLRP3 inflammasome assembly or signaling. If this is not known, it would be worth highlighting this topic as a potentially important future area of investigation in this field.
6. It would also help to add depth to their mini-review article if they at least briefly commented on how things like high-fat diet, metabolic disease, obesity, etc., are potentially thought to influence the ability of lipid composition/biology to shape NLRP3 inflammasome signaling.

Reviewer #2

(Remarks to the Author)
In this review article, the authors have presented an excellent synthesis of the role of lipids and lipid-based post-translational modifications of the NLRP3 as pivotal mechanisms regulating its activation. This review distinguishes itself from the highly crowded field of general inflammasome research and presents a unique aspect of lipid metabolism in regulating NLRP3 sub-cellular localization and activation. These insights into how membrane lipid composition shapes cellular properties and governs NLRP3 localization across different sub-cellular compartments and/or cell type-related properties are highly thought-provoking. They are directly relevant to future translational studies focusing on the lipid-NLRP3 signaling axis. This highly relevant review offers impactful, engaging insights for a broad audience of innate immune and inflammatory researchers.
Specific comments:
• While the review presents excellent figures for quick understanding of the topics discussed, adding a table listing the

known lipids and related metabolites with specific functions in NLRP3 localization and/or activation may help the readers further.
• Whereas the review heavily focused on lipid-NRLP3 connection, a brief description of a couple of other examples, other than NLRP3 inflammasome, may provide additional context to think about the unique and general functions of lipids and lipid rafts in regulating different cell death complexes.
• Adding a short section related to the relevance of lipid-NLRP3 signaling to health and disease may also enhance the impact of the review.

Reviewer #3

(Remarks to the Author)
This review summarizes recent studies on the roles of lipids in the NLRP3 pathway. The topic is increasingly important, and the manuscript provides a solid introduction. I have a few suggestions to improve clarity:
1. Figure citations should indicate the corresponding panels.
2. Figure 2C shows GSDMD cleavage, IL-1β and IL-18 release, and pyroptosis. It would be helpful to incorporate this panel into Figure 1 and cite it in the first paragraph on page 4. NINJ1 should also be included in the figure.
3. The second paragraph on page 4 should include a brief summary of the signals that activate the NLRP3 inflammasome.
4. An additional figure illustrating NLRP3 palmitoylation would be very helpful, particularly to show the modification sites and their proposed functions.

Version 1:

Reviewer comments:

Reviewer #2

(Remarks to the Author)
The comments are addressed.

Open Access This Peer Review File is licensed under a Creative Commons Attribution 4.0 International License, which permits use, sharing, adaptation, distribution and reproduction in any medium or format, as long as you give appropriate credit to the original author(s) and the source, provide a link to the Creative Commons license, and indicate if changes were made.
In cases where reviewers are anonymous, credit should be given to 'Anonymous Referee' and the source.
The images or other third party material in this Peer Review File are included in the article’s Creative Commons license, unless indicated otherwise in a credit line to the material. If material is not included in the article’s Creative Commons license and your intended use is not permitted by statutory regulation or exceeds the permitted use, you will need to obtain permission directly from the copyright holder.

To view a copy of this license, visit https://creativecommons.org/licenses/by/4.0/

We thank the Editors and Reviewers for taking the time to carefully review our manuscript, and for the constructive comments and valuable suggestions provided. We have addressed all Reviewers' comments satisfactorily and revised the manuscript accordingly.

The point-by-point responses to the reviewers' comments are as follows:

Reviewers' comments:

Reviewer #1 (Remarks to the Author):

In this excellent mini-review article, Anand and Aljadeed provide a thoughtful and timely overview of the emerging literature demonstrating that lipid composition, especially at distinct organelles, can profoundly shape NLRP3 inflammasome assembly and signaling. They do a magnificent job of describing relevant molecular details that govern this crosstalk between lipid biology and the NLRP3 inflammasome. In addition, they nicely highlight the significance of this line of work for the design of improved NLRP3 inflammasome-directed therapies. The authors do an especially impressive job of covering recent discoveries in this field, including highlighting high-impact work that was just recently published. They cover the major subject areas in a fair and balanced manner. The figures are very attractive and professionally done, and will serve as a great resource for others in the field. Overall, this is an excellent review article that is extremely well written and organized. Below, I list a few very minor critiques, that once addressed, should help to make this already outstanding review article even better.

We are grateful to the Reviewer for their thoughtful and supportive feedback and are delighted that they found the manuscript informative.

Specific comments:

1. The acronyms ER and TGN should probably be defined in the abstract.
2. It appears that a space is needed between "nucleates" and "ASC" in line 133.
3. They should double-check the citation style in line 175, as it appears to be inconsistent with the numbered approach used throughout the rest of the mini-review article.
4. SREBP2 overlaps with the ER structure in Figure 1, which makes it hard to read. They should consider moving it to improve readability.

Thank you for your suggestions. Points 1-4 have now been incorporated into the revised manuscript at appropriate places.

5. To increase the scope of the mini-review article, it would be interesting to know how lipid oxidization as occurs in ferroptosis, influences NLRP3 inflammasome assembly or signaling. If this is not known, it would be worth highlighting this topic as a potentially important future area of investigation in this field.

We appreciate the Reviewers' suggestion and have highlighted lipid oxidation during ferroptosis in the 'Conclusions and future perspectives' section of the revised manuscript.

6. It would also help to add depth to their mini-review article if they at least briefly commented on how things like high-fat diet, metabolic disease, obesity, etc., are potentially thought to influence the ability of lipid composition/biology to shape NLRP3 inflammasome signaling.

We thank the Reviewer for their recommendation and have added a new section on 'Metabolic disease and lipid-dependent regulation of inflammasome signalling' to the revised manuscript.

Reviewer #2 (Remarks to the Author):

In this review article, the authors have presented an excellent synthesis of the role of lipids and lipid-based post-translational modifications of the NLRP3 as pivotal mechanisms regulating its activation. This review distinguishes itself from the highly crowded field of general inflammasome research and presents a unique aspect of lipid metabolism in regulating NLRP3 sub-cellular localization and activation. These insights into how membrane lipid composition shapes cellular properties and governs NLRP3 localization across different sub-cellular compartments and/or cell type-related properties are highly thought-provoking. They are directly relevant to future translational studies focusing on the lipid-NLRP3 signaling axis. This highly relevant review offers impactful, engaging insights for a broad audience of innate immune and inflammatory researchers.

We sincerely appreciate the Reviewer's encouraging remarks and are pleased that the manuscript was perceived as informative and valuable.

Specific comments:

• While the review presents excellent figures for quick understanding of the topics discussed, adding a table listing the known lipids and related metabolites with specific functions in NLRP3 localization and/or activation may help the readers further.

We are grateful to the Reviewer for their suggestion. We have now added a new table (Table 1) listing the lipids and metabolites and their roles in NLRP3 localisation and activation in the revised manuscript.

• Whereas the review heavily focused on lipid-NRLP3 connection, a brief description of a couple of other examples, other than NLRP3 inflammasome, may provide additional context to think about the unique and general functions of lipids and lipid rafts in regulating different cell death complexes.

Thank you for the suggestion. A new section on ‘Lipid-dependent spatial organization in innate immune signalling’ has been added to the revised manuscript. This section additionally covers the roles of lipids in the activation of STING, necroptosis, and pyroptosis, highlighting a key role for spatial organization in immune signalling.

• Adding a short section related to the relevance of lipid-NLRP3 signaling to health and disease may also enhance the impact of the review.

We thank the Reviewer for their recommendation and have added a new section on ‘Metabolic disease and lipid-dependent regulation of inflammasome signalling’ in the revised manuscript.

Reviewer #3 (Remarks to the Author):

This review summarizes recent studies on the roles of lipids in the NLRP3 pathway. The topic is increasingly important, and the manuscript provides a solid introduction. I have a few suggestions to improve clarity:

1. Figure citations should indicate the corresponding panels.

2. Figure 2C shows GSDMD cleavage, IL-1β and IL-18 release, and pyroptosis. It would be helpful to incorporate this panel into Figure 1 and cite it in the first paragraph on page 4. NINJ1 should also be included in the figure.

3. The second paragraph on page 4 should include a brief summary of the signals that activate the NLRP3 inflammasome.

We thank the Reviewer for their positive comments. The above suggestions have now been incorporated into the revised manuscript at appropriate places.

4. An additional figure illustrating NLRP3 palmitoylation would be very helpful, particularly to show the modification sites and their proposed functions.

We are grateful to the Reviewer for their suggestion. Figure 2A in the manuscript already includes a schematic of NLRP3 highlighting the key palmitoylation sites. To complement this, we have now included a table (Table 2) summarising the known palmitoylation sites and their functions in NLRP3 activation.
